# L5 Nerve Root Radiculopathy as a Rare Complication Following Oblique Lateral Interbody Fusion at L5/S1 Combined With Posterior Fixation via Percutaneous Pedicle Screws for Adult Spinal Deformity

**DOI:** 10.7759/cureus.58969

**Published:** 2024-04-25

**Authors:** Yoshinori Maki, Kenji Fukaya

**Affiliations:** 1 Neurosurgery, Hikone Chuo Hospital, Hikone, JPN; 2 Neurosurgery, Ayabe Renaiss Hospital, Ayabe, JPN

**Keywords:** radiculopathy, adult spinal deformity, l5-s1, complication, oblique lateral interbody fusion

## Abstract

Oblique lateral interbody fusion (OLIF) is an established and less invasive surgical approach for patients with adult spinal deformities. This method can also be applied to the L5/S1 region (termed “OLIF51”); however, reports on L5 nerve root radiculopathy as a rare complication of OLIF51 are limited. Here, we present the case of a 77-year-old woman with progressive adult spinal deformity who was followed up after an initial OLIF for the L3/4 and L4/5 levels. An additional operation was performed to resolve ambulation difficulty and back pain related to adult spinal deformity. Circumferential fixation was performed over two sessions. Initially, OLIF51 was performed concurrently with OLIF for L1/2 and L2/3. Eight days later, posterior fixation surgery from T10 to the ilium via percutaneous pedicle screws was performed. Two days after the second operation, the patient started complaining of left L5 nerve root radiculopathy, for which medication and rehabilitation were both ineffective. Retrospectively, we identified that the left L5/S1 foramen narrowed after the lordotic correction by OLIF51 and posterior fixation.

Additionally, posterior facetectomy for L5/S1 was performed, and the left L5 nerve root radiculopathy was resolved. L5 nerve root radiculopathy can develop as a rare complication of OLIF51. Neurosurgeons should be aware of this rare complication related to OLIF51.

## Introduction

Oblique lateral interbody fusion (OLIF) is an established surgical approach for adult spinal deformity. This surgical approach is typically performed from the L1/L2 to L4/L5 levels. However, due to its low invasive nature and excellent lordotic lumbosacral correction, OLIF is also performed for the L5/S1 level (OLIF51) [1-3]. However, reports concerning perioperative complications potentially related to OLIF51, such as L5 nerve root radiculopathy, are rare. Herein, we describe the first reported case of postoperative L5 nerve root radiculopathy possibly resulting from OLIF51.

## Case presentation

**Figure 1:**
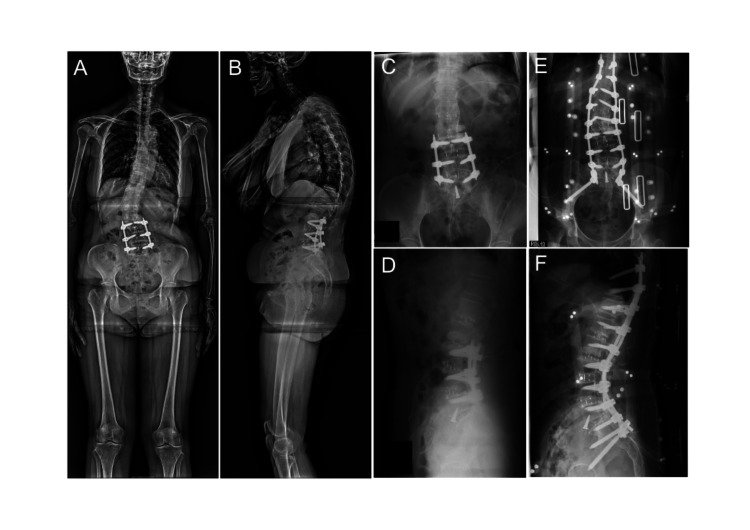
Radiographs before and after oblique lumbar intervertebral fusion and posterior fixation via percutaneous pedicle screws. Adult spinal deformity following the first oblique lumbar intervertebral fusion (OLIF) of L3/4 and L4/5 was observed. The angle between the L5 and S1 was originally 12°, changing to 18° after OLIF, including the L5/S1 level, and 23° after posterior fixation via percutaneous pedicle screws. Images taken (A, B) before OLIF, including the L5/S1 level, (C, D) after OLIF, (E, F) after posterior fixation via percutaneous pedicle screws. (A, C, E) anterior-posterior projection, and (B, D, F) lateral projection.

A 77-year-old woman with a past history of OLIF of the L3/4 and L4/5 presented with back pain and ambulation difficulty related to adult spinal deformity (Figure 1A, 1B). The patient wished to undergo surgical management; therefore, we planned thoracic-lumbar fixation surgery in two sessions. First, OLIF was performed at three levels (L1/2, L2/3, and L5/S1), with the patient set in the right lateral decubitus position. A skin incision of approximately 7 cm was made, and the anterior retroperitoneal space was entered retrogradely. After identifying the sacral promontory, the bilateral iliac vessels were laterally retracted. The anterior longitudinal ligament of the L5/S1 was cut, and the intervertebral disc was replaced with an artificial cage (degree: 12°, size: 32mm × 23mm × 12mm, SOVEREIGN SPINAL SYSTEM, Medtronic). OLIF at the L1/2 (cage degree: 6°, size: 8mm × 40mm × 18mm, Clydesdale PTC, Medtronic) and L2/3 (cage degree: 6°, size: 8mm × 45mm × 18mm, Clydesdale PTC, Medtronic) was successively performed (Figure 1C, 1D). Eight days after OLIF at three levels, posterior fixation surgery was performed from T10 to the ilium using percutaneous pedicle screws (Figure 1E, 1F).

**Figure 2:**
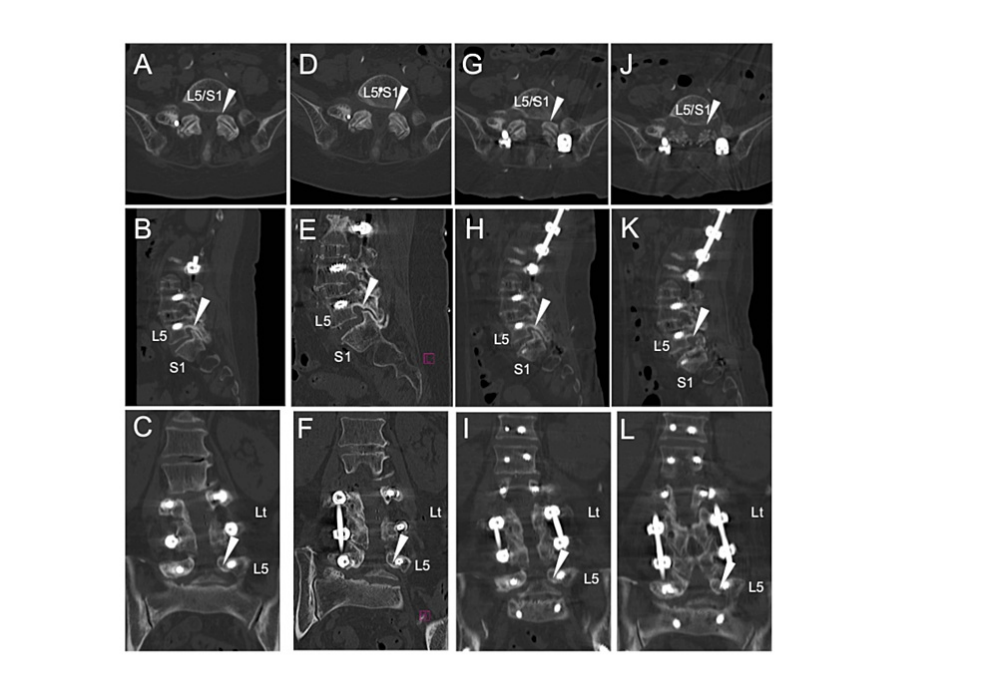
Chronological changes in the right L5/S1 foramen on computed tomography images Following oblique lumbar interbody fusion at the L5/S1, indirect decompression of the same level was temporality achieved (A-F). However, the right L5/S1 foramen (arrowheads) narrowed after posterior fixation surgery (G-I). The stenosis of the L5/S1 foramina was managed with posterior decompression surgery (J-L). Images taken (A-C) before oblique lumbar interbody fusion including the L5/S1 level, (D-F) after oblique lumbar interbody fusion including the L5/S1 level, (G-I) after posterior fixation via percutaneous pedicle screws, (J-L) after posterior decompression of the bilateral L5/S1 foramina. Figures A, D, G, and J show axial images, B, E, H, and K show sagittal images, and C, F, I, and L show coronal images, (Lt: left).

The angle between the L5 and S1 changed chronologically from 12° (before OLIF) to 23° (after the second operation). Two days after the second operation, when the patient started ambulating, she complained of pain radiating from the left buttock through the posterior thigh to the anterior ankle, which corresponded to the dermatome of L5. Medication failed to sufficiently control the patient’s pain. Retrospectively, we observed that the foramen of the left L5/S1 had narrowed after OLIF51 and posterior fixation surgery (Figure 2A-2I). We thought the nerve root was compressed at the level of L5/S1; therefore, we performed posterior facetectomy for the bilateral L5/S1 levels 15 days after the second operation. Surgery was performed on the left side to treat the symptomatic lesion and on the right side to prevent the same complication. The superior articular process intraoperatively compressed the left L5 nerve root. After the decompression operation (Figure 2J-2L), the pain decreased. The patient was discharged 25 days after rehabilitation.

## Discussion

The following adverse events have previously been reported as perioperative complications related to OLIF (including OLIF51): anterior cage displacement, subsidence, postoperative ileus, retrograde ejaculation, superior mesenteric artery syndrome, blood transfusion, and arterial or venous vascular injury [2,4,5]. Of note, as the bilateral iliac arteries, iliac veins, and their branches are located anteriorly near the intervertebral level of L5/S1, particular attention should be paid to avoid vascular complications during OLIF51 [5]. However, to the best of our knowledge, similar cases of L5 nerve root radiculopathy following OLIF51 have not been reported before.

The patient reported here underwent OLIF (at L1/2, L2/3, and L5/S1) and posterior fixation using percutaneous pedicle screws over two sessions. A lordotic change at the L5/S1 after surgery was radiologically confirmed. As the foramen of the L5/S1 narrowed following lordotic correction, the left L5 nerve root was irritated by the superior articular process. This mechanism could have resulted in left L5 nerve root radiculopathy, necessitating additional decompression surgery. Indeed, excellent lordotic correction is considered an advantage of OLIF51 [1-3]. In addition, when treating this patient, we further cut the anterior longitudinal ligament of L5/S1 to insert an artificial cage. The loss of anterior support at the level L5/S1 could have resulted in selecting an artificial cage with a large degree. This condition could have led to the diminished posterior part of the intervertebral space, narrowing the foramen of L5/S1. This feature of OLIF51, combined with the lack of anterior support, may have resulted in an excessive lordotic correction. Surgeons should be aware of this rare complication after OLIF51. To avoid this rare complication, a large artificial disc should not be chosen; otherwise, lordotic correction may result in a foraminal stenosis of L5/S1. However, as this is the first report of L5 nerve root radiculopathy possibly related to OLIF51, the ideal size of the artificial disc for OLIF51 should be assessed following the accumulation of similar cases. In cases where L5 nerve root radiculopathy following OLIF51 occurs, posterior facetectomy could be useful, as in the present case.

## Conclusions

As presented in this case, L5 nerve root radiculopathy can occur as a rare complication after OLIF51. This complication could have resulted from lordotic correction in two-staged surgery. An additional posterior decompression operation may be necessary to resolve this complication in case it is refractory only with medication and/or rehabilitation. Surgeons should be cautious of this adverse event while performing OLIF51.

## References

[1] Chung NS, Jeon CH, Lee HD, Kweon HJ (2017). Preoperative evaluation of left common iliac vein in oblique lateral interbody fusion at L5-S1. Eur Spine J.

[2] Kotani Y, Ikeura A, Saito T (2023). Comparative clinical analysis of oblique lateral interbody fusion at L5/S1 versus minimally invasive transforaminal interbody fusion (MIS-TLIF) for degenerative lumbosacral disorders. Spine Surg Relat Res.

[3] Orita S, Shiga Y, Inage K (2021). Technical and conceptual review on the L5-S1 oblique lateral interbody fusion surgery (OLIF51). Spine Surg Relat Res.

[4] Berry CA, Thawrani DP, Makhoul FR (2021). Inclusion of L5-S1 in oblique lumbar interbody fusion-techniques and early complications-a single center experience. Spine J.

[5] Woods KR, Billys JB, Hynes RA (2017). Technical description of oblique lateral interbody fusion at L1-L5 (OLIF25) and at L5-S1 (OLIF51) and evaluation of complication and fusion rates. Spine J.

